# Preparation and characterization of biopolymer-based adsorbents and their application for methylene blue removal from wastewater

**DOI:** 10.1038/s41598-023-44613-6

**Published:** 2023-10-12

**Authors:** Mohammad Javad Amiri, Maryam Raayatpisheh, Mohsen Radi, Sedigheh Amiri

**Affiliations:** 1 Department of Water Engineering, Faculty of Agriculture, Fasa University, Fasa, 74616-86131 Iran; 2 Department of Food Science and Technology, Yasooj Branch, Islamic Azad University, Yasooj, Iran; 3 Sustainable Agriculture and Food Security Research Group, Yasooj Branch, Islamic Azad University, Yasooj, 75914-93686 Iran

**Keywords:** Pollution remediation, Chemical engineering

## Abstract

In the present study, four biopolymer-based materials consisting of native corn starch (CS), phosphate corn starch (PS), starch nanocrystals (SNCs), and phosphate corn starch nanocrystals (PSNCs) were synthesized and used for methylene blue (MB) removal as a function of various parameters, including initial MB concentration (C0, 10–500 mg L^−1^), adsorbent dosage (Cs, 0.02–0.15 g), contact time (t, 5–15 min), solution pH (2–11), and temperature (25–45 °C). The removal percentage of MB increased dramatically upon increasing the biopolymer dosage, temperature, and pH; while it decreased upon increasing the initial MB concentration. The adsorption behavior of biopolymer-based materials towards MB was found to be accurately described by the pseudo-second-order kinetic and Langmuir isotherm models. According to the Langmuir model, the maximum adsorption capacities of the adsorbents were ordered as follows: PSNCs (88.53 mg g^−1^) > SNCs (79.55 mg g^−1^) > PS (73.17 mg g^−1^) > CS (63.02 mg g^−1^). PSNCs was able to remove 96.8% and 76.5% of 20 mg L^−1^ MB in greywater and petrochemical wastewater, respectively, at an optimum pH of 9 and retained 86.42% of its usability even after five adsorption–desorption cycles. The analysis of the surface charge of the adsorbents before and after MB adsorption, combined with the FTIR spectrum of MB-saturated biopolymer-based materials, provided evidence that electrostatic interactions was the primary mechanism involved in the adsorption of MB. Meanwhile, hydrogen bonding and π–π interactions were found to have a minor contribution to the adsorption process. Based on the results, it can be inferred that PSNCs has promising potential as an adsorbent for the treatment of MB-containing wastewater, owing to its exceptional properties, which include high adsorption capacity, low cost, and applicability for multiple reuses.

## Introduction

The pollution of water bodies by the various dye compounds has been a major environmental problem in developing countries^1^. Many industries such as textile, paper, printing, leather, food, cosmetics, rubber, and pharmacy are the main recognized anthropogenic sources, which produce a high volume of colored wastewater^2,3^. In this context, the release of dye wastewater into the water bodies without effective and efficient treatment methods creates many health and environmental problems^1,4^. Dysfunction of the central nervous system, skin irritation, itchy or blocked noses, sneezing, and sore eyes are the multiple strenuous health problems of dyes on the humans^5,6^. So, the removal of dyes from colored wastewater has attracted great attention for the researchers.

Among the cationic dyes, Methylene blue (3,7-bis(dimethylamino) phenothiazine chloride tetra methylthionine chloride; MB) derived from industrial wastewater discharge of textile, pharmaceuticals, plastics, cosmetics, paper, and food industries has particular importance due to its persistency, non-biodegradability, and toxicity. MB belongs to a family of azo dye chemical class that can cause various adverse effects on human health and the environment^7^. In light of this, development of versatile and efficient method is needed for MB removal from the environment. Removal of MB can be accomplished by several physicochemical technologies, including advanced oxidation processes^8^, membrane filtration^9^, adsorption^10^, photocatalytic degradation^11^, flocculation^12^, biocomposites films^13^, and Fenton reaction^14^. According to the literature, the removal of MB by most of these techniques is uncompleted and expensive which may produce secondary pollutants^15^. The adsorption process can be a simple, eco-friend, low-cost, and high efficiency alternative to the available conventional and modern wastewater treatments for the removal of MB from dye-stuff effluents^15,16^.

Recently, researchers turn their interest in the adsorption process using bio-polymers-based adsorbents such as alginate^17^, chitosan^18^, cellulose^9,13^, and starch^19^ due to their nontoxic nature, relatively low price, renewability, biodegradability, and easily available. Starch, a natural polysaccharide, is the second most abundant renewable and biodegradable material after cellulose that can be obtained from various natural resources^20^. Two glucosidic macromolecules, amylose and amylopectin, are the basic parts of native starch’s semi-crystalline granules^21^. Linear molecules of glucose units in starch, amylose, consist of 99% (1–4) α-d-glycoside bonds and only a small amount of 1% (1–6) α-linkages. However, amylopectin is an extremely branched polymer, with around 95% α (1–4) linkages and 5% α (1–6). Starch also contains a small amount of proteins and lipids^22^.

Recently the application of starch-based products as effective adsorbents for eliminating contaminants and aromatic compounds from wastewater has become prevalent^4,19^. Some poor features of native starch, such as its propensity to retrogradation, hydrophobic properties, insolubility in water at room temperature, poor adsorption ability, and low tensile strength restrict its direct use for industrial purposes^23^. To resolve these drawbacks and limitations, some active groups including amine phosphate, carboxylate, or xanthate can be used in the starch modification^24^, by using esterification, etherification, cross-linking, oxidation, and acid hydrolysis procedures^23^. Starch phosphates are versatile products that have various applications such as an emulsifier, stabilizers, or thickening agents in different food industries^25^. Cross-linked starch phosphates may also be used as absorbents due to the swelling properties that they have^21^. Depending on the desired characteristics, the degree of substitution of phosphate groups (DSP) is variable. Stability of dispersions (e.g. syneresis or retrogradation), solubility, rheomechanical characteristics, and swelling properties are affected by DSP^26^. In order to increase the surface morphology and physiochemical properties of starch, starch nanocrystals (SNCs) have been synthesized via acid treatment, enzyme treatment or nano-precipitation^27^. The surface of SNCs contains active hydroxyl groups, which are easily reacted with other substances. SNCs have a large specific surface area, small particle size as well as strong chemical reactivity, making them a valuable and effective adsorbent^27^. SNCs are semi-crystalline regions of starch granules that can be obtained by disruption of starch particles’ amorphous parts. Mild acid hydrolysis by hydrochloric or sulfuric acid is applied to isolate the crystalline regions of starch granules^28^. There is a theory that as the amorphous regions of the granule are attacked by acid molecules more aggressively than the crystalline regions at temperatures below gelatinization, they are hydrolyzed more rapidly. As a result of acid hydrolysis, starch nanocrystals are formed, which have high crystallinity and a nanoscale platelet morphology^29^.

The goal of the present study is to investigate the adsorption of MB onto native corn starch and its modification forms (corn starch nanocrystals, phosphate starch and phosphate corn starch nanocrystals (PSNCs) in the batch mode. They were characterized by various techniques, including scanning electron microscopy combined with an energy dispersive X-ray (SEM–EDX) as well as a thermal field emission scanning electron microscope (FESEM), X-ray diffraction (XRD), Fourier transform infrared (FTIR), Brunauer–Emmett–Teller (BET) surface area analysis, Thermogravimetric analysis (TGA), and point of zero charge. Moreover, the influences of operational parameters consisting of initial pH value, reaction time, initial MB concentration, temperature, and adsorbent mass were evaluated. Finally, the mechanism of MB removal by starch-based adsorbents was investigated in terms of kinetics, equilibrium, and thermodynamics. This approach stands out from previous research due to the distinctive features of phosphate corn starch nanocrystals, encompassing surface functional groups, a substantial number of active adsorption sites that can potentially enhance adsorption capacity, and particle size. The utilization of a stable immobilized phosphate group on the surface of corn starch nanocrystals facilitates exclusive interactions with MB, facilitated by electrostatic interactions, hydrogen bonding, and ion exchange mechanisms.

## Materials and methods

### Chemicals

Analytical grade chemical reagents (methylene blue, monopotassium phosphate, sulfuric acid, sodium hydroxide, ethanol, and hydrochloric acid) were purchased from Merck Co. Darmstadt, Germany. Dye stock solution was prepared by dissolving 1 g of MB in deionized water, and diluting for desired concentrations.

### Analytical techniques

The morphology and chemical composition of four starch-based adsorbents were characterized by SEM–EDX (TESCAN-Vega 3, USA) as well as FESEM (TESCAN-MIRA3, USA); whereas the crystalline structure of adsorbents was analyzed by XRD (Bruker D8 Advance, Germany) with Cu-Kα radiation (λ = 0.154 nm) in the 2θ range of 10°–100°. Surface functional groups of samples were accomplished by FTIR (PerkinElmer Spectrum two, USA) in the 400–4000 cm^−1^ wave number range. The porous structure of the samples consisting of specific surface area, average pore diameter, and pore volume was measured by nitrogen adsorption–desorption isotherms at 77 K (Belsorp mini II instrument, Japan). The heat stability of samples was examined by thermogravimetric analysis (TGA) (Setaram TG-DTA92, France). The point of zero charge was measured by a zeta potential meter (Zetasizer Nano ZS90, Malvern, UK) over the pH range of 2.0–11.0. The pH of the suspension was measured by a Knick 766 Calimatic pH meter (Germany) and adjusted using 0.1N HCl/NaOH. Initial and final concentrations of MB were measured using UV–Vis spectroscopy (UV-2100 Double Beam, Beijing, China) at 668 nm.

### Preparation of adsorbents

The native corn starch was purchased from Dornesha, Pars Khooshe Pardaz Factory, Iran, and named as CS. In order to prepare corn starch nanocrystals, a certain amount of CS concentration (14.69 wt%/acid) was dispersed in a diluted sulfuric acid solution (3.16 M) in an Erlenmeyer flask, and the dispersion was magnetically stirred (100 rpm) at an isothermal condition 40 °C for 5 days^30^. After diluting the suspension with distilled water, it was centrifuged five times (15 min, 4500 rpm) in order to remove excess sulfuric acid until neutrality. The resultant suspension was subjected to mechanical treatment with a homogenizer for 5 min at 10,000 rpm to break up the aggregates. Finally, successive filtration processes were applied to separate any unhydrolyzed starch or micrometer-size particles through a 2 μm filter paper^31^. The obtained starch nanocrystal solution was dried in an oven at 130 °C, or a few drops of chloroform were added to it to prevent bacterial growth during storage at 4 °C. The obtained adsorbents were named as SNCs. Phosphate starch (PS), was prepared following a previously published method^32^. 100 g of CS along with 30 g of monopotassium phosphate (KH_2_PO_4_) were dissolved in 100 mL of distilled water, and then the obtained mixture was continuously homogenized for half an hour by a magnetic stirrer. The resulting slurry was filtered through a Buchner funnel with 40-µm filter paper and a vacuum pump. Sediments were left at room temperature for 12 h, then they were heated in an oven for 3 h at 150 °C. After cooling, the reaction product was suspended in distilled water for a volume of 1000 mL. The filtration process was continued until the water passing through the filter paper did not become cloudy when calcium chloride solution (1%) was added, which indicates that there were no phosphates present in the water. The final product was dried in an oven at 40 °C for 3 days and denoted as PS. After the extraction of PS, their nanocrystals were also prepared exactly according to the described method and named as PSNCs. In the next step, 75 g of each of the desired adsorbents including SNCs, PS, and PSNCs, were dissolved in distilled water in the Erlenmeyer flasks at a volume of 500 mL. Then, each sample was subjected to ultrasound treatment for 15 min with a 60% oscillation field. After that, it was taken out of the ultrasonic bath for 5 min. Then they were subjected to ultrasonic oscillations again for another 15 min. The temperature of the bath during the process was completely under control (not to exceed 25 °C). Finally, the treated solutions were filtered and dried in an oven.

### Adsorption experiments

Batch-adsorption studies were conducted to study the different environmental factors’ effect on MB adsorption capacity. The effect of environmental factors including initial MB concentration (C_0_, 10–500 mg L^−1^), adsorbent dosage (C_s_, 0.02–0.15 g), contact time (t, 5–15 min), solution pH (2–11), and temperature (298–318 K) that affect MB adsorption were evaluated. The starch-based adsorption capacity at equilibrium (q_e_, mg g^−1^), and at any time (q_t_, mg g^−1^) as well as the adsorption efficiency (AE, %) were calculated using the following equations:

1 $$q_{e} = \frac{{C_{0} - C_{e} }}{m} \times \;V,$$

2 $$q_{t} = \frac{{C_{0} - C_{t} }}{m} \times \;V,$$

3 $$R\% = \frac{{C_{0} - C_{e} }}{{C_{0} }} \times 100,$$

where C_0_, C_e_, and C_t_ are the liquid-phase concentrations of MB at initial, equilibrium and at any time (mg L^−1^), respectively, m is starch-based adsorbent mass (g), and V is MB solution volume (L). Each experiment was replicated three times and the average values were reported. To determine the interactions between the MB and the bio-polymers-based adsorbents and the adsorption rate constants, three adsorption kinetic models (i.e. pseudo-first-order, pseudo-second-order, and intra-particle diffusion models) were fitted to the contact time-dependent experiments data as follow^33–35^:

4 $$pseudo{\text{-}}first{\text{-}}order\,model:\;q_{t} = \;q_{e} (1 - e^{{ - k_{1} t}} ),$$

5 $$pseudo{\text{-}}second{\text{-}}order\,model:\,q_{t} = \;\frac{{q_{e}^{2} k_{2} t}}{{1 + q_{e} k_{2} t}},$$

6 $$intra{\text{-}}particle \, diffusion \, model:\,q_{t} = \;k_{i} t^{0.5} + \;C,$$

where $k_{1}$ (min^−1^), $k_{2}$ (g mg^−1^ min^−1^) and $k_{i}$ (mg g^−1^ min^−0.5^) represent the pseudo-first-order, pseudo-second-order, and intra-particle diffusion rate constants, respectively, $q_{e}$ and $q_{t}$ (mg g^−1^) are the equilibrium adsorption capacities at equilibrium and at any time, respectively, $C$ and $t\;(\min )$ are the intercept of intra-particle diffusion model and contact time, respectively. In order to determine the capacity of the bio-polymers-based adsorbents for MB adsorption, equilibrium isotherms were studied in terms of Freundlich, Langmuir, and Langmuir–Freundlich. These models can be expressed as^34,36^:

7 $$Langmuir\,model:\,q_{e} = \frac{{k_{L} q_{M} C_{e} }}{{\left( {1 + k_{L} C_{e} } \right)}},$$

8 $$Freundlich\,model:\,q_{e} = k_{F} C_{e}^{1/n},$$

9 $$Langmuir{-}Freundlich\;model:\;q_{e} = \frac{{k_{LF} q_{MLF} C_{e}^{1/b} }}{{\left( {1 + k_{LF} C_{e}^{1/b} } \right)}},$$

where $C_{e}$ $\left( {{\text{mg}}\;{\text{L}}^{ - 1} } \right)$ is the equilibrium concentration of MB in solution, $k_{L}$ $\left( {{\text{L}}\;{\text{mg}}^{ - 1} } \right)$, and $k_{F}$ $\left( {\left( {{\text{mg}}\;{\text{g}}^{ - 1} } \right)\;\left( {{\text{L}}\;{\text{mg}}^{ - 1} } \right)^{1/n} } \right)$ are the Langmuir and Freundlich isotherm constants representing the adsorption strength and adsorption capacity, respectively, $k_{LF}$ $\left( {{\text{L}}\;{\text{mg}}^{ - 1} } \right)^{\frac{1}{n}}$ is the Langmuir–Freundlich adsorption isotherm constant, $q_{M}$ $({\text{mg}}\;{\text{g}}^{ - 1} )$ and $q_{MLF}$ $({\text{mg}}\;{\text{g}}^{ - 1} )$ are the Langmuir and Langmuir–Freundlich maximum adsorption capacity, respectively, $\frac{1}{n}$ (dimensionless) and $\frac{1}{b}$ (dimensionless) are the Freundlich and Langmuir–Freundlich heterogeneity constant, respectively. The thermodynamic parameters such as the changes in the Gibbs free energy $(\Delta G^{ \circ } )$, enthalpy $(\Delta H^{ \circ } )$, and entropy $(\Delta S^{ \circ } )$ were calculated in the range from 298 to 318 K to determine the effect of temperature on MB adsorption by bio-polymers-based adsorbents^36^:

10 $$\Delta G^{o} = - RT\ln k_{d} ,$$

11 $$\Delta G^{ \circ } = \Delta H^{ \circ } - T\Delta S^{ \circ } ,$$

12 $$\ln k_{d} = \frac{{\Delta S^{ \circ } }}{R} - \frac{{\Delta H^{ \circ } }}{RT},$$

13 $$k_{d} = 1000\;k_{g} d,$$

where $k_{d}$ (dimensionless) is the thermodynamic equilibrium constant, $R$ is the gas constant $(8.314\;{\text{J mol}}^{ - 1} {\text{K}}^{ - 1} )$, $T$ is the temperature $({\text{K}})$, $k_{g}$ $({\text{L mg}}^{ - 1} )$ is determined from the isotherm model that is multiplied by 1000 to be converted into $({\text{L g}}^{ - 1} )$, $d$ is the density of water^37^. The applicability of kinetic and isotherm models was verified through the determination coefficient $(R^{2} )$ and Chi-square statistic test $(\chi^{2} )$ as follows^36^:

14 $$R^{2} = \frac{{\sum\limits_{i = 1}^{n} {\left[ {\left[ {(q_{e} )_{\bmod el} - (\overline{{q_{e} }} )_{\bmod el} } \right]\left[ {(q_{e} )_{\exp } - (\overline{{q_{e} }} )_{\exp } } \right]} \right]} }}{{\sum\limits_{i = 1}^{n} [ (q_{e} )_{\exp } - (\overline{{q_{e} }} )_{\exp } ]^{2} \sum\limits_{i = 1}^{n} {\left[ {(q_{e} )_{\bmod el} - (\overline{{q_{e} }} )_{\bmod el} } \right]^{2} } }},$$

15 $$\chi^{2} = \sum\limits_{i = 1}^{i = N} {\frac{{(q_{e,\exp } - q_{e,\bmod el} )^{2} }}{{q_{e,\bmod el} }}} ,$$

where $q_{e,\exp }$ $({\text{mg}}\;{\text{g}}^{ - 1} )$ and $q_{e,\bmod el}$ $({\text{mg}}\;{\text{g}}^{ - 1} )$ are the equilibrium uptake capacity acquired experimentally and in modeling, respectively, $(\overline{{q_{e} }} )_{\exp }$ and $(\overline{{q_{e} }} )_{\bmod el}$ are the average of the experimental and predicted equilibrium adsorption capacity, respectively. A larger $R^{2}$ value along with a smaller $\chi^{2}$ value shows the better performance of the model.

### Desorption and regeneration experiments

To evaluate the reusability of the bio-polymers-based adsorbents, adsorption/desorption experiments were conducted for five consecutive cycles using 30% acetic acid solution^38^. The suitability of acetic acid as a desorption agent for MB is attributed to its acidic nature that induces electrostatic repulsion between MB and the bio-polymers-based materials surface, resulting in the release of MB from the adsorbent. For each cycle, 0.1 mg of the bio-polymers-based adsorbents were added to 100 mL of 10 mg L^−1^ MB solution at optimum pH (pH 9), agitation period of 15 min, and agitation rate of 400 rpm. Then the MB-loaded bio-polymers-based particles were washed with 50 mL of 30% acetic acid solution and the percentage of desorption was calculated as follows^39^:

16 $$Percentage\;of\;desorption\;(\% ) = \frac{\;released\;MB\;concentration}{{initially\;adsorbed\;MB\;concentration}} \times 100.$$

## Results and discussion

### Characterization of the biopolymer-based adsorbents

The BET and BJH methods were employed to determine the specific surface area, while the total pore volume and mean pore diameter were calculated according to the BJH equation. The BET_Surface area_ of CS, PS, SNCs, and PSNCs were found as 2.63, 2.67, 2.91, and 3.13 m^2^ g^−1^, respectively, while The BJH_Surface area_ of those were calculated as 7.67, 8.33, 8.78, and 9.15 m^2^ g^−1^, respectively. Adsorption isotherm patterns of the samples could be categorized as linear according to the IUPAC classification (see Fig. S1). The total pore volumes of CS, PS, SNCs, and PSNCs were found to be 0.0087, 0.0093, 0.0099, and 0.01 cm^3^ g^−1^, respectively, and the average pore diameters of those were calculated as 10.1, 9.1, 8.7, and 8.3 nm, respectively (see Fig. S1). So, the pore structures of biopolymer-based adsorbents have mesoporous structures because the pores diameter are between 2 and 50 nm^40^.

**Figure 1:**
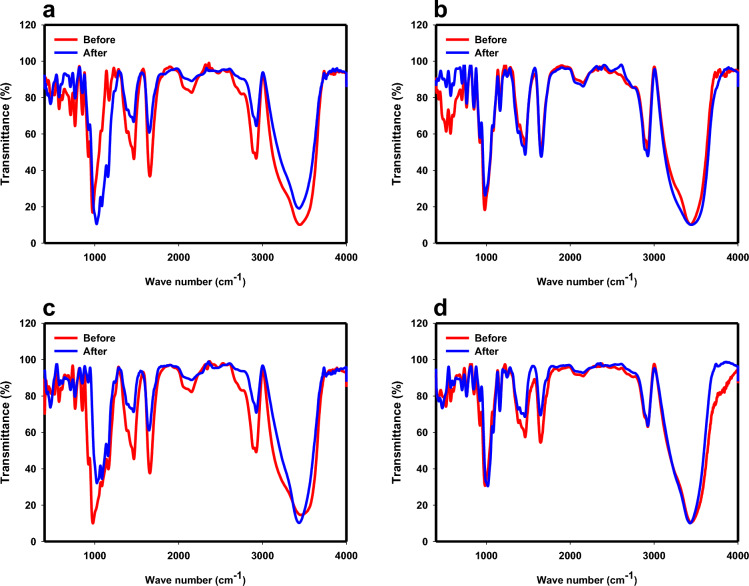
The FTIR spectra of samples: (**a**) CS, (**b**) PS, (**c**) SNCs, and (**d**) PSNCs before and after MB adsorption.

The FTIR spectra of CS, PS, SNCs, and PSNCs are shown in Fig. S2, which are very similar to each other. The broad peaks at about 3437–3456 cm^−1^ were attributed to O–H stretching vibration of the amylopectin^34^. The strong adsorption peaks at about 2923–2925 cm^−1^ were assigned to the C–H stretching in the alkane group. Other peaks at about 1646–1656 cm^−1^ were contributed to the H–O–H bending from water molecules^28^, C=C stretching vibration and N–H bending^41^. The strong peaks in the region 1461–1466 cm^−1^ can be assigned to C–N stretching vibration of amide group and aromatic C=C bond stretching, which show the present of polymer chain in all four samples^42^. The weak peaks in the structures of CS, PS, and PSNCs at about 1160–1254 cm^−1^ were associated with C–N and C–O, which disappeared for SNCs^41^. The peaks at about 974–984 cm^−1^ as well as 852 cm^−1^ and 765 cm^−1^ were ascribed to the C–O band stretching^19^. The FTIR spectrum of the PS, and PSNCs shows that a new peak at about 518–520 cm^−1^ formed during the phosphate stabilization. The FTIR spectrum of the biopolymer-based adsorbents after interaction with MB solution indicates that no new peaks formed during the MB adsorption (see Fig. 1). However, a decreasing and slight shifting in characteristic absorbance peaks in the FTIR spectrum of biopolymer-based adsorbents, particularly for CS, SNCs, and PSNCs, after MB adsorption occurred. The peaks of aromatic C=C bonds decreased in intensity and shifted after MB adsorption, except PS adsorbent, which elucidated the presence of π-π interaction between MB and biopolymer-based adsorbents^43^. In the case of PS, an electrostatic interaction might occur between the positive charge of MB and the $P{O}_{4}^{-}$ present on the PS surface^44^.

**Figure 2:**
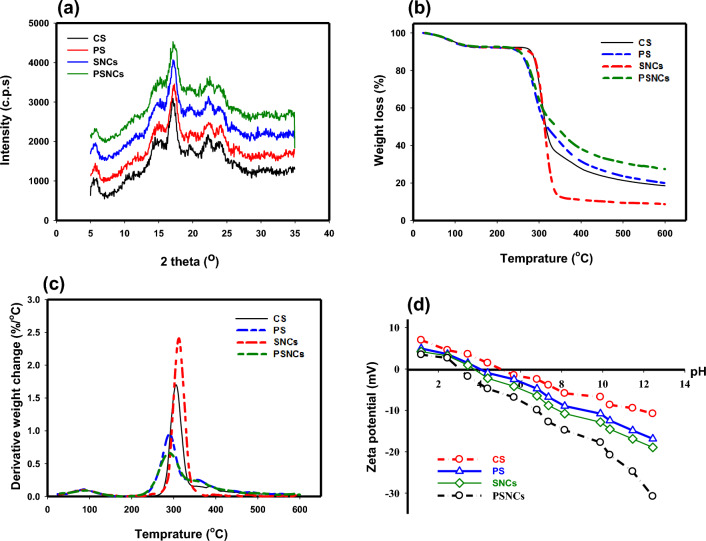
(**a**) XRD; (**b**) TG; (**c**) DTG, and (**d**) zeta potential of biopolymer-based adsorbents at different pH values.

The XRD patterns of CS, PS, SNCs, and PSNCs samples are presented in Fig. 2a. The results indicated that the main peaks of biopolymer-based adsorbents are similar. The four main diffraction peaks at 2Ө = 15.4, 17.5, 23.3, and 24.3° are attributed to the high degree of crystallinity of starch^45^. However, the diffraction peaks with higher intensity in the modified starch structures indicated that the crystallinity value was increased due to the action of sulfonic acid and phosphate groups^41^.

The thermal stability of CS, PS as well as their nanocrystals (SNCs, and PSNCs) was determined using thermo-gravimetric analysis (TGA) (Fig. 2b). Then differential thermal gravimetric (DTG) curves obtained from the TGA-temperature profiles were calculated (Fig. 2c). As temperature increased, the TGA curve displayed a reduction in weight, in three distinct stages. Primarily, this phenomenon is attributed to the dehydration and evaporation of unstable elements in the samples. In the second range, it results from the depolymerization of starch, and in the final stage, it is due to the polymer’s backbone decomposition^46^. The slight variations between CS and SNCs indicated that the depolymerization process of SNCs is initiated earlier in comparison to CS. This is because of the existence of sulfate groups situated on the surface of SNCs which act as catalysts for the reaction^47^. However, according to Xu et al. the observed reduction in the thermal decomposition temperature of SNCs subsequent to acid hydrolysis may be attributed to their tightly packed crystalline structure and enhanced intermolecular bonding. This is due to the fact that the degradation of starch is caused by dehydration reactions between water molecules and either the inter- or intramolecular components of the starch molecules, with water serving as the principal decomposition product. As a result, the more compact crystalline structure resulting from acid hydrolysis led to a faster rate of this decomposition reaction^31^. The amylopectin section of starch contains natural phosphate molecules that are covalently linked to glucose units. In PS, the amount of bound phosphorus can be increased by augmenting the number of accessible sources for hydroxyl groups. The graph of PS demonstrates that at high temperatures there is less weight loss, possibly attributable to the abundance of inorganic components they possess^32^.

To analyze the value of the charge repulsion/attraction between the bio-polymers-based adsorbents and MB, the zeta potential of the samples as a function of solution pH is measured and illustrated in Fig. 2d. The point of zero charge (pH_ZPC_) for CS, PS, SNCs, and PSNCs was found to be 5, 4, 3.7, and 3, respectively. At pH < pH_ZPC_, the surface charge of the samples becomes positive, while it turns to negative at pH > pHZPC. The modified starches show a lower pH_ZPC_ due to the introduction of sulfonic acid groups by acid hydrolysis or the presence of phosphate group after phosphorylation. It was found that the zeta potential gradually decreased when the solution pH increased from 1 to 12. When the pH was 12, the zeta potential of CS, PS, SNCs, and PSNCs reached the maximum value of − 10.8, − 16.8, − 18.9, and − 30.78 mV, respectively, thus resulting in higher electrostatic attraction between MB and bio-polymers adsorbent surface.

**Figure 3:**
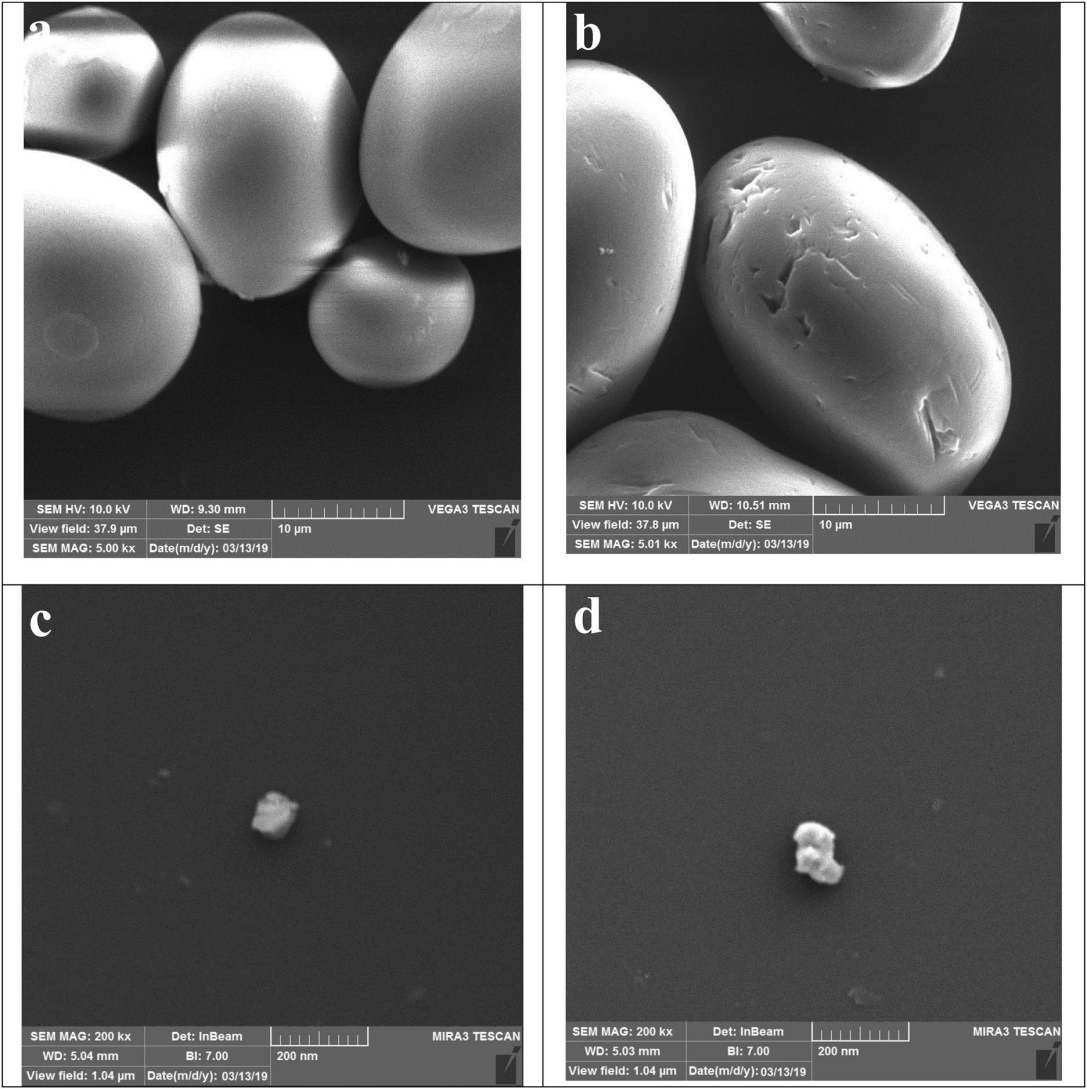
SEM analysis of CS (**a**), PS (**b**), and FESEM analysis SNCs (**c**) and PSNCs (**d**).

SEM images of CS typically reveal that the individual granules exhibit a spherical or near-spherical shape, appearing as rounded structures with smooth surfaces (Fig. 3a). In comparison to CS, the granules of PS exhibit an uneven surface with noticeable surface irregularities (Fig. 3b). The introduction of phosphate groups through chemical modification can induce alterations in the surface morphology of CS. FESEM images of SNCs and PSNCs typically depict individual particles with rough and irregular surfaces, often characterized by a faceted or angular structure (Fig. 3c,d). EDX analysis was conducted on randomly selected regions to examine the chemical composition of the bio-polymers-based materials. Based on the measurements, carbon and oxygen are identified as the primary elements of CS, comprising 57.84% and 42.16% of the total weight, respectively. Following phosphorylation, the surface of the PS displays a discernible phosphorus signal, representing 7.32% of the overall composition. After the hydrolysis of CS with sulfuric acid, an elemental sulfur content of 9.16% was detected. The phosphorus and sulfur contents in PSNCs were measured to be 7.86% and 9.23%, respectively.

### Effect of operational factors on MB adsorption

**Figure 4:**
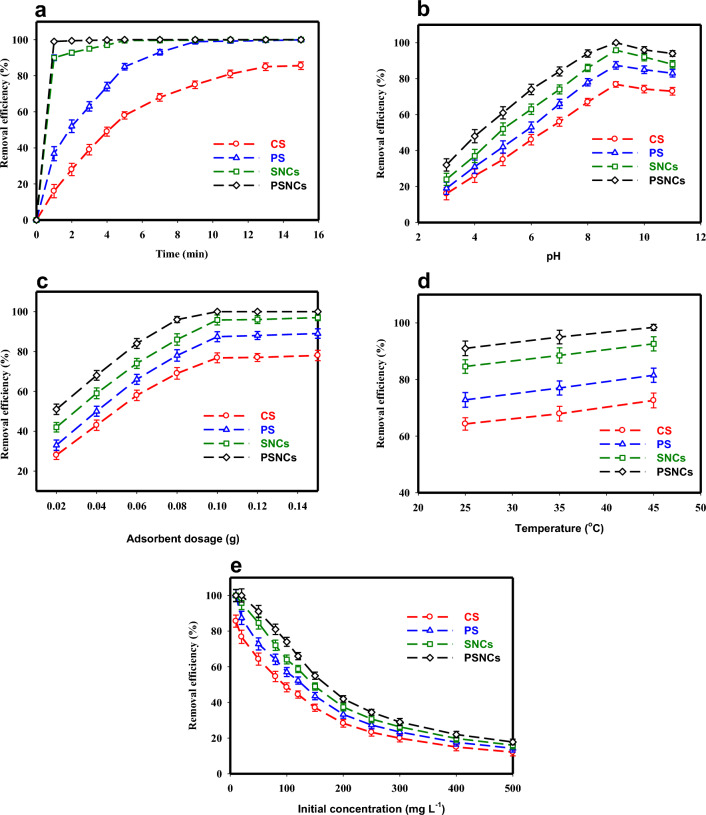
Effects of (**a**) contact time (temperature, 25 ± 1 °C; MB concentration, 10 mg L^−1^; pH, 7; adsorbent dosage, 0.1 g), (**b**) pH (temperature, 25 ± 1 °C; MB concentration, 20 mg L^−1^; contact time, 15 min; adsorbent dosage, 0.1 g), (**c**) adsorbent dose (temperature, 25 ± 1 °C; MB concentration, 20 mg L^−1^; contact time, 15 min; pH, 9), (**d**) temperature (MB concentration, 50 mg L^−1^; contact time 15 min; pH, 9; adsorbent dosage, 0.1 g) and (**e**) initial concentration (temperature, 25 ± 1 °C; contact time, 15 min; pH, 9; adsorbent dosage, 0.1 g) on MB adsorption.

The adsorption efficiency of MB with an initial concentration of 10 mg L^−1^ by 0.1 g bio-polymers-based adsorbents as a function of contact time (0–15 min) is illustrated in Fig. 4a. According to this Figure, MB was rapidly adsorbed onto the bio-polymers-based adsorbents due to the presence of numerous vacant adsorption sites, and equilibrium was reached within 1, 5, 10, and 15 min for PSNCs, SNCs, PS, and CS respectively. It was observed that PSNCs had a substantially faster reaction rate with MB compared to the other adsorbents under the same condition, resulting in the complete removal of MB (100%) by PSNCs within just 1 min. The results suggest that the modification of starch would greatly improve its reaction rate, which is consistent with prior research studies^19,28^.

The adsorption efficiency of MB by CS, PS and their nanocrystals (SNCs and PSNCs) was significantly affected by the pH of the solution. The pH level of the solution can alter both the surface charge of the bio-polymers-based adsorbents and the ionization state of the MB^48^. Figure 4b illustrates the impact of solution pH on the adsorption efficiency of bio-polymers-based adsorbents at an initial pH between 3 and 11. The removal efficiency of MB significantly increased as the solution pH increased from 3 to 9, but then decreased again as the pH was further increased from 9 to 11. The optimal pH for achieving the highest adsorption efficiency was found to be 9 and selected for the rest of this study. The impact of pH in MB adsorption can be attributed to the pH_ZPC_ and the surface protonation and deprotonation phenomena of the bio-polymers-based adsorbents^49^. When the pH is lower than pH_ZPC_, the bio-polymers-based adsorbent surface acquires a positive charge and becomes protonated by the presence of H^+^ ions. This leads to an increase in electrostatic repulsion between the MB positive ions and the positively charged surface of the bio-polymers-based adsorbent. Consequently, the removal efficiency of the adsorbents decreases. When the pH is higher than pH_ZPC_, the presence of OH^−^ ions deprotonates the carboxyl groups on the surface of the bio-polymers-based adsorbents and leading to the formation of carboxylate. So, the –COOH groups on the adsorbents lose H^+^ ions and the negatively charged –COO^−^ ions are formed. As a result, there is an increase in electrostatic interactions as well as ionic bonding between the MB positive ions and the negatively charged sites on the bio-polymers-based adsorbents, which increases the adsorption efficiency of the adsorbent for dye removal^44,48,49^. At more alkaline condition (pH > 9), the removal efficiency of MB decreases due to the structural changes in MB. As shown in Fig. 4b, the nanocrystals of both native and phosphate starches possessed a greater capacity for adsorption. This may be due to the emergence of sulphate groups on the surface of nanocrystals following acid hydrolysis. These negatively charged groups can then bind with MB through electrostatic forces, thereby facilitating the process of adsorption^28^. In this study, the PSNCs compared to the other three adsorbents, had a higher level of reactivity with MB in the entire range of pH.

The influence of adsorbent dosage on MB removal was investigated at seven dosages (0.02, 0.04, 0.06, 0.08, 1, 0.12, and 0.15 g) when the initial concentration of MB (20 mg L^−1^), pH (9), and contact time (15 min) were kept constant (Fig. 4c). According to Fig. 4c, an improvement in the removal efficiency of MB was observed for all four adsorbents when the adsorbent dosage was increased from 0.02 to 0.1 g. This can mainly be owing to an augmentation in either the number of active sites or the surface area available for interaction between MB and adsorbents, leading to an increase in the contact between MB molecules and bio-polymers-based adsorbents^50^. Despite increasing the dosage of each adsorbent beyond 0.1 g, there was no significant change in the adsorption efficiency and the removal rate of the adsorbent gradually reached a plateau. This could be attributed to the achievement of the equilibrium adsorption capacity at higher dosages of adsorbent.

The influence of temperature on the adsorption efficiency of MB was examined at three values (25, 35 and 45 °C) when the initial concentration of MB (50 mg L^−1^), pH (9), and contact time (15 min) were kept constant (Fig. 4d). According to the findings, there was a considerable improvement in dye removal efficacy by adsorbents as the temperature increased from 25 to 45 °C. This can be attributed to the higher number of available adsorption sites and greater mobility of MB at elevated temperatures^3^. Furthermore, an increase in temperature can lead to the expansion and enlargement of small pores, allowing larger dye molecules to become accessible^2^.

Figure 4e illustrates the effect of MB’s initial concentration on the adsorption efficiency at a dosage of 0.1 g in an optimized pH solution of 9 after 15 min contact time. When the initial concentration of MB increased from 10 to 500 mg L^−1^, the removal efficiency of PSNCs, SNCs, PS, and CS decreased significantly from 100–17.8%, 100–16%, 99.9–14.24% to 85.6–12.1%, respectively. It was found that further increasing of initial concentration had no considerable improvement in adsorption efficiency. The high removal efficiency of MB at low concentrations can be related to the more surface area and vacant active sites. At higher concentrations, the adsorbent-to-solution ratio remains constant, leading to saturation of the adsorbent's exchangeable sites^51^.

### Adsorption kinetics, isotherms, and thermodynamics

Table 1 presents the kinetic parameters and variables that were fitted to three common kinetic models (i.e. pseudo-first-order, pseudo-second-order, and intra-particle diffusion models) for the adsorption of MB. According to the results, the pseudo-second-order model (R^2^ = 99.53–99.99; $\chi^{2}$ = 0.009–0.11) provided a better fit to the experimental data compared to the pseudo-first-order (R^2^ = 92.15–99.11; $\chi^{2}$ = 0.018–0.12) and intra-particle diffusion (R^2^ = 70.41–96.58; $\chi^{2}$ = 0.027–0.74) models. This indicates that the MB adsorption process is significantly influenced by chemisorption. Furthermore, the calculated adsorption capacity obtained from the pseudo-second-order model were found to be in excellent agreement with the corresponding experimental values (Table 1). The second-order rate constants (k_2_) for MB adsorption followed the order of PSNCs (0.695 g mg^−1^ min^−1^) > SNCs (0.688 g mg^−1^ min^−1^) > PS (0.038 g mg^−1^ min^−1^) > CS (0.012 g mg^−1^ min^−1^). This order indicates that PSNCs has a faster adsorption process than other adsorbents under identical experimental conditions. Based on the obtained results, the plots of the intra-particle diffusion model were linear; however, they did not intersect at the origin. It was found that intra-particle diffusion, particularly for CS and PS adsorbents with relatively high R^2^ values, participates in the adsorption process but it is not the rate-determining step^40^.

**Table 1:** Corresponding kinetic parameters for the adsorption of MB onto bio-polymers-based adsorbents.

Adsorbent	Model	Kinetics parameters	Value	R^2^	$\chi^{2}$
CS	Pseudo-first-order	*k*_1_ (min^−1^)	7.73	99.11	0.082
		*q*_*e*_ (mg g^−1^)	11.94		
	Pseudo-second-order	*k*_2_ (g mg^−1^ min^−1^)	0.012	99.87	0.056
		*q*_*e*_ (mg g^−1^)	8.75		
	Intraparticle diffusion	*k*_*i*_ (mg g^−1^ min^−0.5^)	2.52	96.58	0.38
		*C*	0.41		
PS	Pseudo-first-order	*k*_1_ (min^−1^)	2.27	98.78	0.12
		*q*_1_ (mg g^−1^)	11.76		
	Pseudo-second-order	*k*_2_ (g mg^−1^ min^−1^)	0.038	99.53	0.11
		*q*_2_ (mg g^−1^)	10.56		
	Intraparticle diffusion	*k*_*i*_ (mg g^−1^ min^−0.5^)	2.22	88.24	0.74
		*C*	2.51		
SNCs	Pseudo-first-order	*k*_1_ (min^−1^)	0.133	92.15	0.02
		*q*_1_ (mg g^−1^)	10.08		
	Pseudo-second-order	*k*_2_ (g mg^−1^ min^−1^)	0.688	99.99	0.009
		*q*_2_ (mg g^−1^)	10		
	Intraparticle diffusion	*k*_*i*_ (mg g^−1^ min^−0.5^)	0.327	77.39	0.027
		*C*	8.92		
PSNCs	Pseudo-first-order	*k*_1_(h^−1^)	0.012	95.07	0.018
		*q*_1_ (mg g^−1^)	10.06		
	Pseudo-second-order	*k*_2_ (g mg^−1^ min^−1^)	0.695	99.99	0.009
		*q*_2_ (mg g^−1^)	10		
	Intraparticle diffusion	*k*_*i*_ (mg g^−1^ min^−0.5^)	0.03	70.41	0.038
		*C*	9.9		

**Figure 5:**
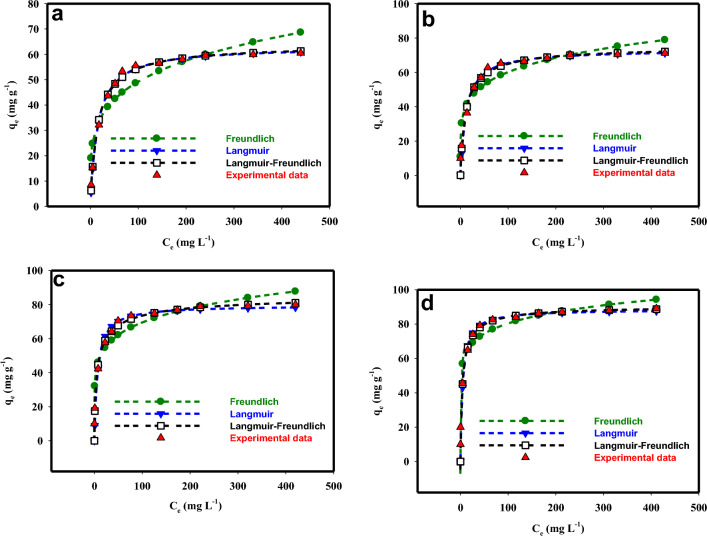
The measured and predicted equilibrium data by Langmuir, Freundlich, and Langmuir–Freundlich isotherm models for MB adsorption onto (**a**) CS, (**b**) PS, (**c**) SNCs, and (**d**) PSNCs.

The diagrams of the MB adsorption isotherm model by bio-polymers-based materials are depicted in Fig. 5 and the corresponding parameters of the adsorption isotherms are listed in Table 2. According to the obtained R^2^ and standard error of estimate (SEE) values, the Langmuir model (R^2^ = 95.12–99.54; SEE = 1.26–6.31) is the best-fitting isotherm model for the adsorption of MB by bio-polymers-based materials, followed by Langmuir–Freundlich (R^2^ = 93.94–99.46; SEE = 1.36–7.1), and Freundlich (R^2^ = 89.48–93.58; SEE = 5.87–7.69) models. Furthermore, the adsorption capacity obtained from the Langmuir model is consistent with the experimental values presented in Table 2. The agreement of the equilibrium data with the Langmuir isotherm model demonstrates that the adsorption follows a monolayer process. Similar results have been reported by Mouni et al., Basaleh et al. and Saxena et al., demonstrating that the equilibrium data of MB adsorption by Kaolin, polyamide-vermiculite nanocomposites, and functionalized multiwalled carbon nanotubes, respectively, followed Langmuir isotherm model due to monolayer adsorption. The maximum adsorption capacity (q_m_) for MB adsorption by PSNCs (88.53 mg g^−1^) obtained from the Langmuir isotherm model was significantly higher than that of SNCs (79.55 mg g^−1^), PS (73.17 mg g^−1^), and CS (63.02 mg g^−1^). The results obtained from the BET, SEM, and FTIR analyses strongly corroborated the findings of the isotherm and kinetic studies conducted on PSNCs. The observed high second-order rate constants and enhanced adsorption capacity can be attributed to the presence of functional groups such as hydroxyl (–OH), carboxyl (–COOH), sulfate (–SO42–), and phosphate (–PO4–), as evidenced by FTIR analysis. Furthermore, the BET analysis demonstrated that PSNCs possess a larger surface area and a greater number of available adsorption sites, further supporting their increased adsorption capacity. Additionally, the SEM analysis revealed that PSNCs exhibit a small particle size and surface roughness, which likely contribute to their favorable adsorption properties. Collectively, these results provide compelling evidence for the role of functional groups, surface area, and particle characteristics in the enhanced adsorption behavior of PSNCs.

**Table 2:** The parameters of the adsorption isotherms for MB onto the bio-polymers-based adsorbents.

Model	CS	PS	SNCs	PSNCs	Rank
Langmuir					1
*q*_*m*_ (mg g^−1^)	63.02	73.17	79.55	88.53	
*K*_*L*_ (L mg^−1^)	0.066	0.085	0.15	0.21	
R^2^	99.54	97.88	98	95.12	
SEE	1.26	3.21	3.52	6.31	
Langmuir–Freundlich					2
*K*_*L*_ (L mg^−1^)^1/*n*^	0.076	0.118	0.281	0.284	
1/*n*	0.938	0.865	0.648	0.828	
*q*_*m*_ (mg g^−1^)	63.95	75.32	86.8	90.79	
R^2^	99.46	97.5	97.11	93.94	
SEE	1.36	3.53	4.25	7.1	
Freundlich					3
1/*n*	0.224	0.185	0.161	0.112	
*K*_*F*_ $\left( {\left( {{\text{mg}}\;{\text{g}}^{ - 1} } \right)\;\left( {{\text{L}}\;{\text{mg}}^{ - 1} } \right)^{1/n} } \right)$	17.55	25.64	33.06	48.03	
R^2^	89.48	92.93	93.58	92.75	
SEE	6.03	5.87	6.34	7.69	

*SEE* standard error of estimate.

As depicted in Fig. 4d, the adsorption capacity of bio-polymers-based materials increased as the temperature of MB solutions was raised from 25 to 45 °C. Table 3 lists the values of ΔH° and ΔS° obtained from the slope and y-axis intercept, respectively, of the plot of InK_d_ versus 1/T, as shown in Fig. S3. At all the temperatures investigated, ΔH° and ΔS° are positive, while ΔG° is negative. The positive value of ΔH° as well as the negative value of ΔG° suggested that the MB adsorption process by bio-polymers-based materials was a spontaneous endothermic reaction. In addition, the positive value of ΔS° suggests an increase in randomness at the solid/solution interface during the MB adsorption^36^. An increase in the negative values of ΔG° was observed upon increasing the temperature from 25 to 45 °C, suggesting that the MB adsorption becomes more energetically favorable. In addition to the sign of ∆H°, the magnitude of this value can provide information about whether the adsorption process is physisorption or chemisorption. The range of ∆H° values between 2.1 and 20.9 kJ mol^−1^ is classified as physisorption, while chemisorption is associated with ΔH° values in the range of 20.9 to 418.4 kJ mol^−1^. The values of ΔH° calculated for CS and PS were 15.17 kJ mol^−1^ and 19.6 kJ mol^−1^, respectively, falling within the range of physisorption. In contrast, the values for SNCs and PSNCs were 32.35 kJ mol^−1^ and 70.86 kJ mol^−1^, respectively, followed chemisorption mechanism. PSNCs exhibits a higher affinity for MB adsorption compared to other adsorbents, as evidenced by its greater values of ΔH° and ΔS°, and more negative values of ΔG°. According to the literature^52^, ΔG° values up to 20 kJ mol^−1^ have been linked to electrostatic interactions. On the other hand, ΔG° values more negative than 40 kJ mol^−1^ indicate the occurrence of charge sharing from the surface of bio-polymers-based materials to the dye molecules, resulting in the formation of a coordinate bond. Based on the data presented in Table 3, the calculated values of ΔG° for CS, PS, PSNCs, and SNCs were all below 20 kJ mol^−1^. These values fall within the range typically associated with physisorption. These findings are in line with the kinetic and equilibrium results obtained. The agreement between the equilibrium data and the Langmuir model demonstrates that the adsorption of MB on bio-polymers-based materials involves a combination of physical and chemical processes. The agreement between the experimental data and the pseudo-second-order kinetic model suggests that the rate-limiting step for the adsorption of MB on the bio-polymers-based materials is chemisorption. By considering the knowledge derived from the kinetic, equilibrium, and thermodynamic analyses, it can be inferred that the adsorption of MB on bio-polymers-based materials is attributed to the coexistence of chemisorption and physisorption processes.

**Table 3:** Thermodynamic parameters for MB adsorption by bio-polymers-based materials.

Adsorbent	ΔH° (kJ mol^−1^)	ΔS^o^ (J mol^−1^ K^−1^)	T (K)	ΔG° (kJ mol^−1^)
CS	15.17	55.7	298	− 1.42
			308	− 1.98
			318	− 2.53
PS	19.6	73.82	298	− 2.39
			308	− 3.13
			318	− 3.87
SNCs	32.35	122.54	298	− 4.16
			308	− 5.39
			318	− 6.61
PSNCs	70.86	256.36	298	− 5.53
			308	− 8.09
			318	− 10.66

### Desorption, and recycling

**Figure 6:**
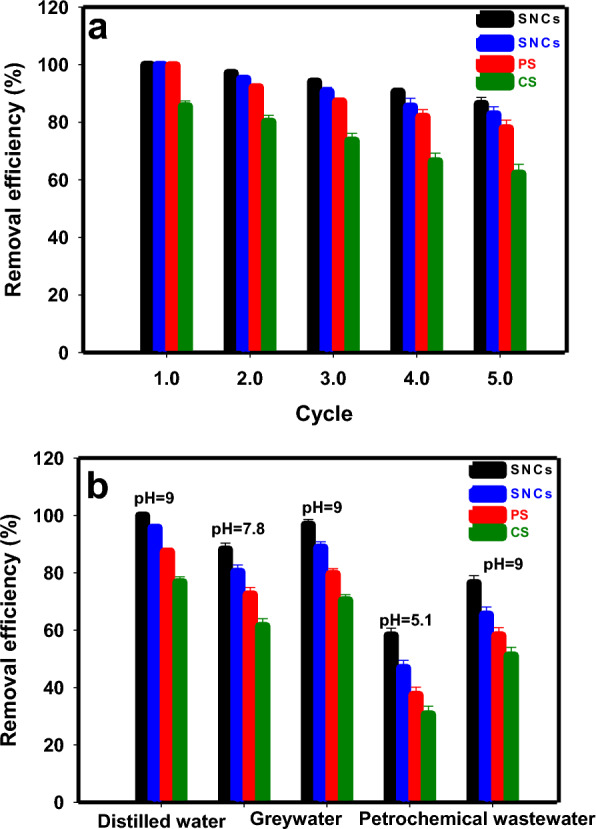
(**a**) The influence of the number of recycling cycles of biopolymer-based materials on the removal efficiency of MB (pH, 9.0, adsorption time, 15 min, adsorbent dosage, 0.10 g, MB concentration, 10 mg·L^−1^ and temperature, 25 ± 1 °C); (**b**) removal efficiency of MB from real water samples after exposure to biopolymer-based materials.

For the economic and practical utilization of adsorbents, desorption and recycling capacity studies are crucial. Figure 6a illustrates the influence of the number of bio-polymers-based materials recycling cycles on the removal rate of MB. The bio-polymers-based materials exhibit high efficiency and stability during adsorption–desorption processes, as efficient recovery and reuse of PSNCs, SNCs, and PS can be attained with only a slight reduction of 13.6%, 17.2%, and 21.9%, respectively, in adsorption efficiency after five cycles. The adsorption efficiency of MB by PSNCs decreased from 100% in the initial cycle to 86.42% in the fifth cycle. The decrease in adsorption efficiency of MB by increasing in the adsorption–desorption cycle might be attributed to several factors. These include: (1) The acetic acid eluent can lead to the protonation of specific adsorption sites or functional groups located on the surface of the bio-polymers-based materials^53^; (2) gradual saturation of the available active sites by MB molecules in the bio-polymers-based materials^53^, and (3) progressive blocking of pores by impurities present in the bio-polymers-based materials^7^.

### Real wastewater application

A batch experiment was conducted to evaluate the performance and effectiveness of bio-polymers-based materials in treating two distinct samples of real wastewater: greywater and petrochemical wastewater. The greywater sample used in the study was collected from the student hostel located at Fasa University in Fars, Iran. The greywater sample had a chemical oxygen demand (COD) of 180 mg L^−1^, a total organic carbon (TOC) of 67.5 mg L^−1^, a biochemical oxygen demand (BOD_5_) of 125 mg L^−1^, and a pH of 7.8. In contrast, the petrochemical wastewater sample had a COD of 350 mg L^−1^, a TOC of 135 mg L^−1^, a BOD_5_ of 220 mg L^−1^, and a pH of 5.1. Following filtration of the real water solution using a 0.45 μm filter membrane, 0.1 g of bio-polymers-based materials was introduced into 100 mL of wastewater that contained 20 mg L^−1^ MB. Then, the adsorption efficiency of MB in the real water sample was measured over a period of 15 min without pH adjustment. Figure 6b illustrates the differences in the effectiveness of bio-polymers-based materials for MB adsorption between optimized conditions in distilled water and real wastewater where the optimized conditions were not utilized. These data indicate that the MB removal efficiency was comparatively lower in petrochemical wastewater than in greywater. In fact, the interference of complex organic compounds presents in the petrochemical wastewater could be a plausible explanation for inhibit adsorption. Furthermore, the lower adsorption efficiency observed in petrochemical wastewater could be also attributed to the low pH of the solution (pH 5.1). The abundance of H^+^ ions in the solution caused the functional groups in the bio-polymers-based materials to undergo protonation, leading to electrostatic repulsion with MB. This finding further corroborates the results obtained from the pH_ZPC_ measurements. The bio-polymers-based materials exhibited the highest performance in treating distilled water, greywater, and petrochemical wastewater in the following order: PSNCs > SNCs > PS > CS. When PSNCs was used to remove MB in greywater, and petrochemical wastewater without any adjustment to the pH, the removal efficiencies were found to be 88.19%, and 58.2%, respectively (Fig. 6b). The removal efficiencies of MB in greywater and petrochemical wastewater were enhanced to 96.8% and 76.5%, respectively, upon adjusting the pH of the dye solution to 9 during treatment with PSNCs.

### Comparison with other adsorbents

Table 4 presents a performance comparison of the PSNCs with those reported in the literature, indicating that it performs comparably or even better in most cases. So, numerous advantages of PSNCs including high adsorption efficiency in spite of a relatively small surface region, non-toxicity, the ability to be regenerated and reused multiple times, and inexpensive manufacturing and usage costs makes it suitable for large-scale MB treatment applications.

**Table 4:** The adsorption capacities for various adsorbents used for MB elimination as compared with this study.

Adsorbent	Conditions	Adsorption capacity (mg g^−1^)	References
Kaolin	pH 6 Temperature = 25 °C Dosage = 0.5 g L^−1^ C_0_ = 10 mg L^−1^	52.76	Mouni et al.^48^
Lemongrass leaves bio-waste (LLB)	pH 9 Contact time = 45 min Temperature = 25 °C C_0_ = 600 mg L^−1^	43.15	Zein et al.^7^
CA-LLB	pH 10 Temperature = 75 °C Contact time = 75 min C_0_ = 1400 mg L^−1^	122.1	Zein et al.^7^
Functionalized MWCNTs	Temperature = 25 °C C_0_ = 300 mg L^−1^ Contact time = 7 min pH 6	440	Saxena et al.^54^
Polyamide-vermiculite nanocomposites	pH 10 C_0_ = 80 mg L^−1^ Contact time = 60 min	76.42	Basaleh et al.^50^
Polymer modified by esterified starch	pH 9 Contact time = 300 min C_0_ = 300 mg L^−1^	62.52	Mu et al.^55^
Starch biocryogel	C_0_ = 50 mg L^−1^ pH 5.1 Temperature = 25 °C	34.84	Taweekarn et al.^34^
Succinylated-starch nanocrystals	pH 9 Contact time = 60 min C_0_ = 200 mg L^−1^	84	Chen et al.^28^
Cellulose acetate nanofibrous membranes modified by polydopamine	pH 6.5 Contact time = 30 h Temperature = 25 °C	88.2	Cheng et al.^9^
Copper nanoparticles	pH 6 Dosage = 1.25 g L^−1^ Temperature = 22 °C	64	Sebeia et al.^56^
Hybrid nanocomposites of carbon nanotube and graphene materials	Dosage = 0.5 g L^−1^ Contact time = 120 min C_0_ = 40 mg L^−1^	24.81	Athari et al.^16^
PSNCs	C_0_ = 500 mg L^−1^ pH 9 Contact time = 15 min	88.53	This study

### Removal mechanism

**Figure 7:**
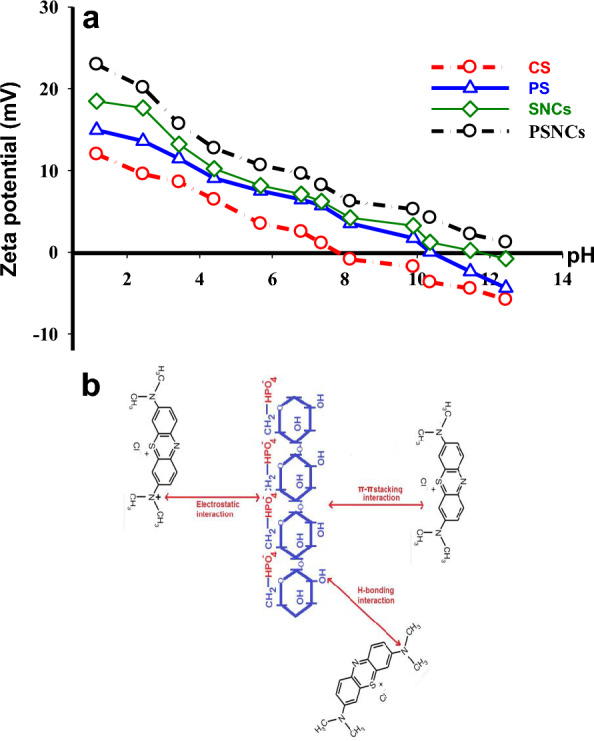
(**a**) Zeta potential of biopolymer-based materials at various pH values after MB adsorption, (**b**) possible adsorption mechanisms of MB onto PSNCs.

A combination of the electrostatic and non-electrostatic interactions between MB and the available active sites present on the bio-polymers-based material’s surface may be involved in the adsorption process. The non-electrostatic interactions consisting of van der Waals forces, hydrophobic interactions, and π–π stacking, may be influenced by several factors, such as the chemical composition and morphology of the bio-polymers-based material’s surface, as well as the molecular structure of the MB^49^. MB can be effectively accumulated on the surface of bio-polymers-based material at pH 9 due to the deprotonation of functional groups such as hydroxyl (–OH), carboxyl (–COOH), sulphate (–SO_4_^2−^), and phosphate (–PO_4_^−^). Increasing the total amount of anionic PO_4_^−^ and SO_4_^2−^ groups in modified starches can lead to a higher number of active sites that are available for MB binding. Figure 7a displays the zeta potentials of bio-polymers-based materials after MB adsorption. Before MB adsorption, the pH_ZPC_ values of CS, PS, SNCs, and PSNCs were 5, 4, 3.7, and 3, respectively (Fig. 2d). However, following MB adsorption, there was a significant increase in the pH_ZPC_ of CS, PS, and SNCs, with values shifting to 7.8, 10.35, and 11.67, respectively. It is worth noting that the zeta potential of PSNCs remains positive across all pH ranges. This can be attributed to the accumulation of positively charged MB adsorbed onto the bio-polymers-based materials. The results demonstrate that electrostatic attraction plays a crucial role in the adsorption of MB onto the bio-polymers-based material’s surface. The adsorption of MB by bio-polymers-based materials involves not only electrostatic attraction, but also other forces such as H-bonding and π–π interactions, which may also play a significant role in this sorption process. The peaks observed at 3443, 3438, 3456, and 3438 cm^−1^ for CS, PS, SNCs, and PSNCs, respectively, suggest a shift in O–H stretching to 3436, 3442, 3435, 3427 cm^−1^ (see Fig. 1). This shift indicates the possibility of hydrogen bonding interaction between the hydrogen of –OH group in the biopolymer-based materials and nitrogen in MB^54,55^. The presence of π–π interaction between MB and biopolymer-based adsorbents was evident from the decreased intensity and shifted peaks of aromatic C=C bonds after MB adsorption, except for the PS adsorbent, as reported by Tran et al. Figure 7b presents the proposed adsorption mechanisms of MB onto PSNCs.

### Column study

**Figure 8:**
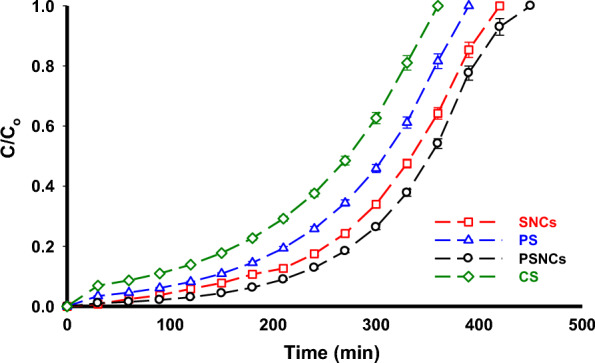
The performance of biopolymer-based materials in a fixed-bed column system.

Figure 8 illustrates the breakthrough profiles obtained in a fixed-bed column system to assess the dynamic behavior of MB adsorption onto the surface of biopolymer-based materials. To accomplish this objective, the experimental conditions were set as follows: a constant initial concentration of MB at 10 mg L^−1^, a flow rate of 5 mL min^−1^, a bed height of 6 cm, and a pH value of 9. Based on the information presented in Fig. 8, it is evident that modifying starch resulted in a rightward shift of the breakthrough profile. Additionally, the breakthrough times for the different materials followed the order of PSNCs > SNCs > PS > CS. In fact, modifying starch can induce various alterations in its adsorption capacity, surface properties, mass transfer properties, and pore structure. These changes, taken together, can cause the breakthrough curves to shift to the right, signifying a prolonged breakthrough time.

## Conclusion

Four biopolymer-based materials, with the capability for rapid separation, recovery, and efficient recycling, were synthesized and effectively utilized for the removal of MB from wastewater. Characterization techniques have revealed that the presence of hydroxyl (–OH), carboxyl (–COOH), sulphate (–SO_4_^2−^), and phosphate (–PO_4_^−^) groups in the adsorbents contribute to enhancing the electrostatic interaction, hydrogen-bonding, π–π interactions between biopolymer-based materials and MB. CS, PS, SNCs, and PSNCs achieved MB removal rates of 100%, 100%, 99.9%, and 85.6% within agitation and mixing durations of 1, 5, 10, and 15 min, respectively. Under the optimal conditions (temperature: 45 °C, MB concentration: 500 mg·L^−1^, pH: 9.0, adsorbent dosage: 1 g L^−1^, adsorption time: 15 min), CS, PS, SNCs, and PSNCs exhibited adsorption capacities of 88.53 mg g^−1^, 79.55 mg g^−1^, 73.17 mg g^−1^, and 63.02 mg g^−1^, respectively. Analysis of the thermodynamic parameters revealed that the MB adsorption was feasible, endothermic and spontaneous. Overall, the unique properties of PSNCs, such as its abundance, renewability, cost-effectiveness and high adsorption capacity, making it a promising and sustainable solution for treating MB-containing wastewater. The production of PSNCs can be readily scaled up, allowing for its potential in the treatment of dye-stuff effluents on an industrial scale.

### Supplementary Information

Supplementary Figures.

## Data Availability

All data generated or analyzed during this study are included in this published article and its Supplementary Information files.
