# Supplementary material for: Preparation and characterization of biopolymer-based adsorbents and their application for methylene blue removal from wastewater
Source: Sci Rep. 2023 Oct 12;13:17263. doi: 10.1038/s41598-023-44613-6 (PMC10570327; doi:10.1038/s41598-023-44613-6)
Supplement: Supplementary file 1 — Supplementary Figures. [file 41598_2023_44613_MOESM1_ESM.docx]

**Supplementary data**

**Preparation and characterization of biopolymer-based adsorbents and their application for methylene blue removal from wastewater**

Mohammad Javad Amiri^1^*, Maryam Raayatpisheh^2^, Mohsen Radi^2,3^*, Sedigheh Amiri^2,3^

^1^ Department of Water Engineering, Faculty of Agriculture, Fasa University, Fasa, 74616-86131, Iran

^2^ Department of Food Science and Technology, Yasooj Branch, Islamic Azad University, Yasooj, Iran

^3^ Sustainable Agriculture and Food Security Research Group, Yasooj Branch, Islamic Azad University, Yasooj, Iran; msnradi@gmail.com (Mohsen Radi); s.amiri@iauyasooj.ac.ir. (Sedigheh Amiri)

*Correspondence: Mohammad Javad Amiri, Email: mj_amiri@fasau.ac.ir; Mohsen Radi, Email: msnradi@gmail.com

|  |  |
| --- | --- |
|  |  |
|  |  |
|  |  |

Figure S1. The N_2_ adsorption–desorption isotherm and pore size distribution of CS (a, b), PS (c, d), SNCs (e, f) and PSNCs (g, h) samples.


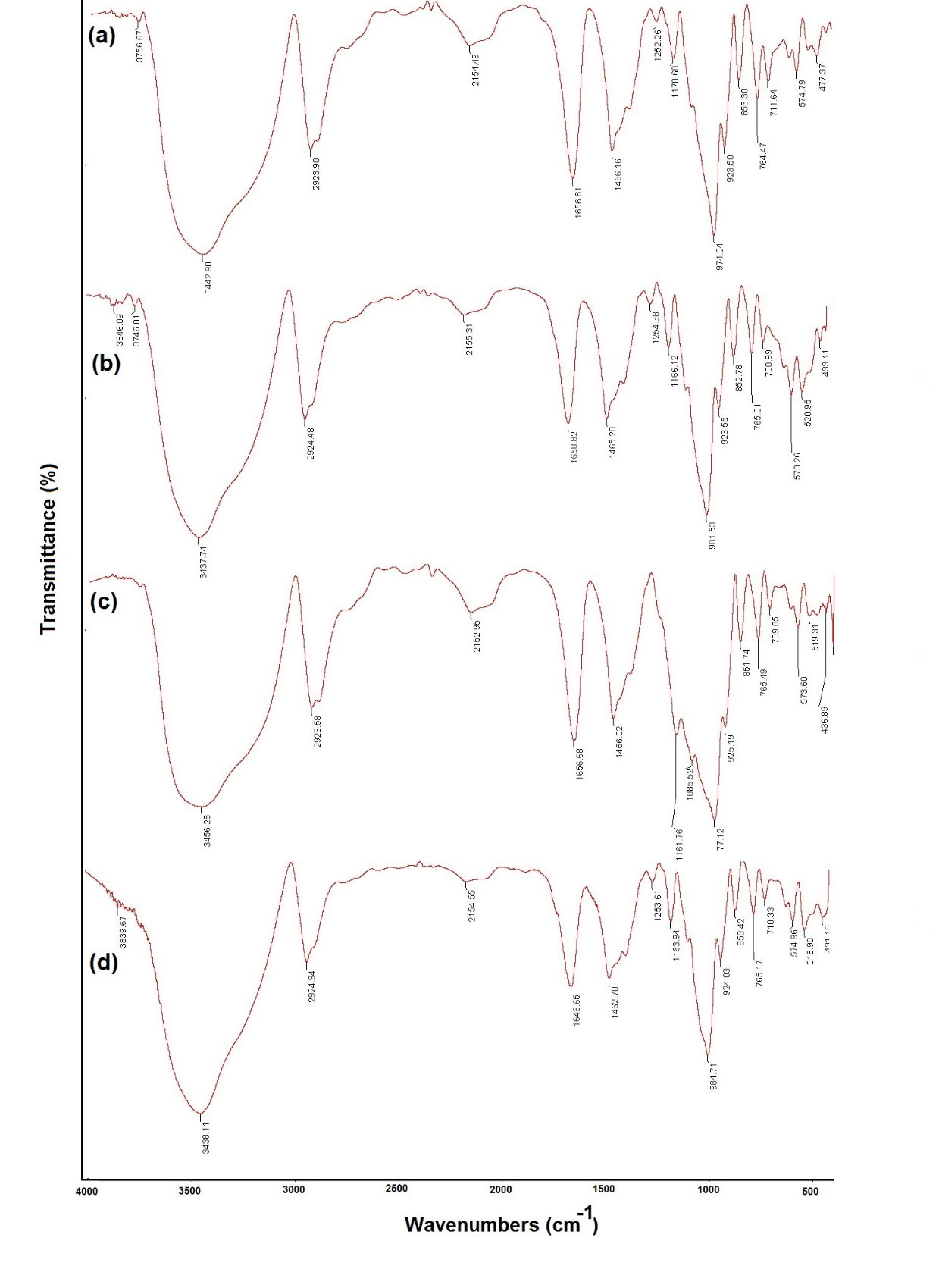


Figure S2. FTIR spectra of (a) CS, (b) PS, (c) SNCs, and (d) PSNCs.

|  |  |
| --- | --- |
|  |  |

Figure S3. Plot of ln(Kd) versus 1/T for the adsorption of MB by (a) CS, (b) PS, (c) SNCs, and (d) PSNCs.
